# Establishment and engineering application of viscoelastic-plastic constitutive laws for creep modeling in interbedded rock masses

**DOI:** 10.1038/s41598-023-48003-w

**Published:** 2023-11-24

**Authors:** Taotao Hu, Shaojun He, Zhibin Kang, Peng Tu, Dong Wang

**Affiliations:** 1 School of Highway, Chang’an University, Xi’an, 710064 China; 2 School of Highway, Chang’an University, Xi’an, 710064 China; 3 China Jiangxi International Economic and Technical Cooperation Co., LTD, Nanchang, 330083 China; 4 School of Energy and Building Engineering, Shandong Huayu University of Technology, Dezhou, 253000 China

**Keywords:** Civil engineering, Mathematics and computing

## Abstract

In order to study the creep behavior of the surrounding rock of the interbedded rock mass tunnel considering the time-dependent deformation, this paper proposes a viscoelastic-plastic seven-element model considering the stress threshold, and derives and establishes its creep equation under three-dimensional stress state. At the same time, the UMAT (User-defined Material) subroutine of the model is developed based on the ABAQUS software. The rationality of the seven-element model and the effectiveness of the subprogram are verified by rheological test results. Finally, the UMAT subroutine is applied to the numerical simulation of the creep behavior of soft and hard interbedded rock tunnels with different rock inclinations (*α*). The results show that the different rock inclination angles have different effects on the horizontal displacement of the ground above the tunnel, settlement deformation, and the convergence of the tunnel section. With the increase of the rock inclination (0 ≤ *α* ≤ 90°), the horizontal displacement of the surface on both sides is antisymmetric. When *α* is 0°, 45° and 90°, the horizontal displacement on both sides is equivalent. Surface subsidence decreases and then increases slowly. When *α* is 0° and 45°, the surface subsidence is the largest (12.4 mm) and the smallest (11.1 mm), respectively. The convergence values of the tunnel section change according to different parts of the tunnel. The convergence values of the arch top and arch bottom decrease continuously, and their maximum convergence values are 23.4 mm and 17.3 mm, respectively. The change trend of the arch waist and arch shoulder convergence values is the opposite. When *α* is 0°, the convergence value of the arch waist is maximum (3.5 mm). When *α* is 15°, the convergence value of the arch shoulder is the maximum (2.0 mm).

## Introduction

Rock rheology is one of the important causes of aging deformation and instability failure in tunnel engineering, mining engineering and slope engineering^1–4^. In recent years, with the rapid development of China's construction engineering technology and the continuous expansion of construction scale, more and more tunnel projects that are deeply buried and pass through poor geological conditions have been put into construction. For deeply buried tunnels with weak interlayers, there are two main challenges: First, deep buried tunnels are often accompanied by high ground stress geological conditions, tunnel excavation is easy to cause the rheological effects of surrounding rock. Second, the weak rock mass has obvious creep behavior in the early stage of support, which brings great difficulties to the tunnel support and operation and maintenance^5–7^.

For the creep characteristics of rock masses, scholars have conducted a lot of research. Significant results have been achieved in theoretical research and engineering practice^8–10^. How to select the appropriate rheological model according to the creep characteristics of rock masses is an important content of rock rheological research. The rheological model of rock mainly includes element combination model and damage-based integral model^11,12^, among which the combination model composed of the basic elements of Newtonian body, Hooke's body and Saint–Venant's body is the most widely used. There are many constitutive models that describe the creep deformation of rock masses, such as the Kelvin model, Burgers model, Nishihara model, etc^13–15^. However, most of them can only describe the deceleration creep and steady creep stage of rock mass creep, but cannot describe the accelerated creep of rock mass. In terms of studying the creep characteristics of soft and hard interbedded rock masses, Ding et al. established a composite viscoelastic-plastic model (Cvisc model) by studying creep failure characteristics of different types of soft and hard rocks, which can effectively simulate the unloading process and rheological behavior of rock mass^16^. Pan et al. conducted a secondary development of the UMAT subroutine based on the finite element theory format of the generalized Nishihara viscoelastic-plastic rheological model, and verified the correctness of the secondary development model program through uniaxial compression creep test^17^. Xiong et al. took interbedded rock mass composed of marble and greenschist as the research object and conducted numerical simulation of uniaxial compression creep test^18^, finding that when the volume content of marble interbedded layer is the same, the failure strength of axial load perpendicular to the bedding is greater than that of axial load parallel to the bedding. Ding et al. used similar materials to make soft and hard interlayered rock samples, and conducted triaxial compression tests on them^19^. It was found that when the dip angle of the interlayer was between 0° and 45°, the strength of the rock sample showed a gradually decreasing trend. Huang et al. studied the failure mechanism and mechanical characteristics of soft and hard interbedded rock masses using different rock dip angles and designing different layer thickness ratios^20^. Although domestic and foreign scholars have made some achievements in the creep characteristics of soft and hard interbedded rock mass, they rarely consider the influence of the strain state on the accelerated creep characteristics, so that the creep characteristics cannot accurately reflect the creep characteristics shown in various stages of the creep test process.

Therefore, a viscoelastic-plastic constitutive relationship considering stress threshold based on the Burgers model to describe the creep characteristics of interbedded rock mass was proposed. Then, the secondary development of the numerical program was completed by combining with the UMAT subroutine of ABAQUS finite element software. The correctness and effectiveness of the program were verified through creep testing. Finally, based on the viscoelastic-plastic numerical program, a numerical model of interbedded rock mass tunnel excavation was established to study the influence of rock inclination on horizontal ground displacement, uneven settlement and convergence around the tunnel. At the same time, the creep behavior law of deep buried tunnel excavation considering the time effect is obtained. This study has certain guiding significance for the support of deep-buried tunnels and the maintenance of its full life cycle, and also provides the feasibility for the application of viscoelastic-plastic constitutive model theory of rock mass in engineering practice.

## Viscoelastic-plastic rheological model

### Characteristics of viscoelastic-plastic rheological model

**Figure 1:**
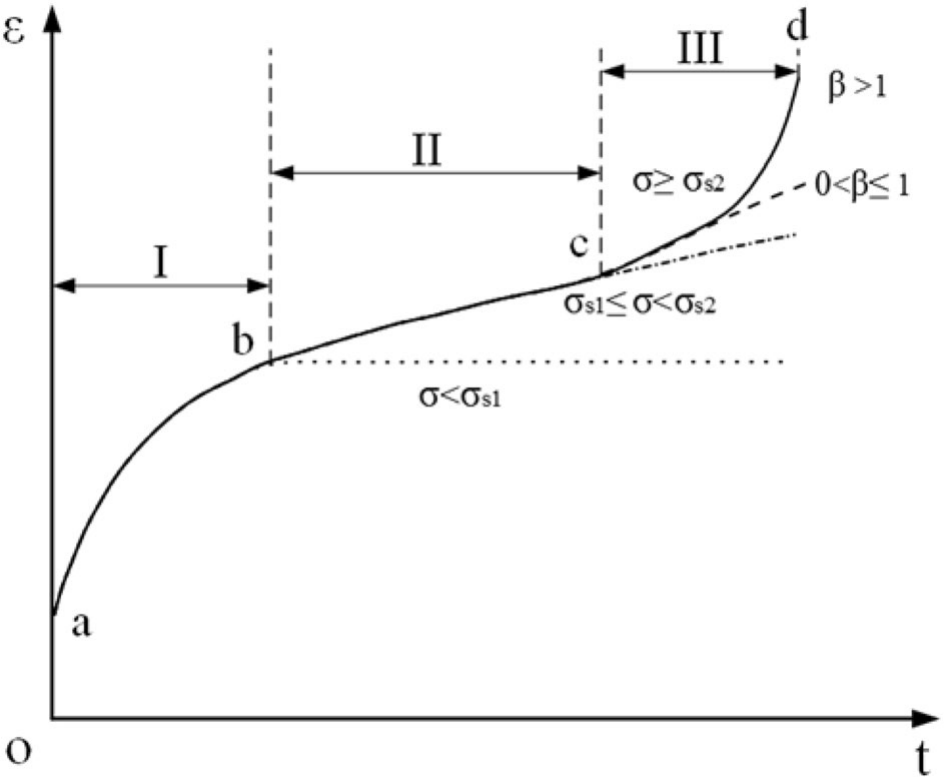
Creep characteristic curve of rock mass.

Generally, a complete rock mass, especially a soft rock mass, has obvious characteristics of time-dependent deformation under higher stress conditions, that is, the rheological properties of the rock mass^21^. The creep of a rock mass with rheological properties is divided into three stages: decelerating creep I (ab), steady-state creep II (bc) and accelerated creep III (cd), as shown in Fig. 1. Reference^16^ shows that the rheological properties of soft and hard interbedded rock masses are significantly different. The creep deformation of soft rock includes three stages of decelerating creep, steady-state creep and accelerated creep, which conform to the creep characteristic of the viscoelastic-plastic constitutive model. The creep deformation of softer rock layers includes two stages, deceleration creep and steady-state creep, which are consistent with the creep characteristics of the Burgers model. While the creep deformation of hard rock layers only has one stage of deceleration creep, which conforms to the creep characteristic of the generalized Kelvin model. It can be seen that compared to a single complete rock mass, the soft and hard interbedded rock mass has higher requirements for the component combination model.

**Figure 2:**
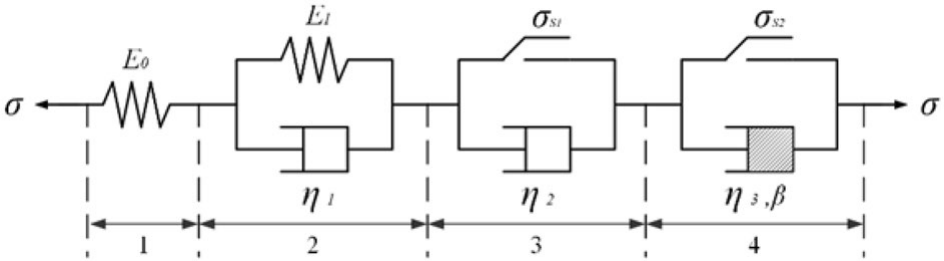
Seven-element viscoelastic-plastic model. *E*_0_ is the instantaneous elastic modulus; *E*_1_ is the average elastic modulus of the rock sample during the decay creep stage; $\eta_{1}$ is the viscosity coefficient, used to indicate the speed of creep during the process of rock attenuation and creep; $\eta_{2}$ is also the viscosity coefficient, which can be used to describe the speed of creep during the steady-state creep stage; $\eta_{{3}}$ is the viscosity coefficient, which can be used to describe the speed of creep during the accelerated creep stage; *β* is the regression coefficient.

Based on the rheological and mechanical properties of soft hard interbedded rock masses, this paper proposes a combination model of viscoelastic-plastic elements considering stress threshold, as shown in Fig. 2. The model can fully reflect the corresponding stages of creep deformation of soft and hard interbedded rock mass, and the combined model should meet the following conditions:

When ${\varvec{\sigma}} < {\varvec{\sigma}}_{s1}$ and ${\varvec{\sigma}} < {\varvec{\sigma}}_{s2}$, all components of parts 1 and 2 in Fig. 2 participate in creep work. The combined model is a three-element model (H–H/N), which can describe the creep characteristics of hard rock. The corresponding state equation can be expressed as

1 $$ \left\{ \begin{aligned} {\varvec{\sigma}}_{0} & = E_{0} {\varvec{\varepsilon}}_{0} \\ {\varvec{\sigma}}_{1} &= {\varvec{E}}_{1} {\varvec{\varepsilon}}_{1} + {\varvec{\eta}}_{1} \frac{{\user2{d\varepsilon }_{1} }}{{{\varvec{dt}}}} \\ {\varvec{\sigma}} &= {\varvec{\sigma}}_{0} = {\varvec{\sigma}}_{1} \\ \varepsilon \;{\mkern 1mu} &= \varepsilon_{0} + \varepsilon_{1} \\ \end{aligned} \right. $$

According to Eq. (1), the one-dimensional creep equation of the three-element model is

2 $$ {\varvec{\varepsilon}}(t) = \frac{\sigma }{{E_{0} }} + \frac{\sigma }{{E_{1} }}\left( {1 - e^{{ - \frac{{E_{1} }}{{\eta_{1} }}t}} } \right) $$

When ${\varvec{\sigma}} \ge {\varvec{\sigma}}_{s1}$ and ${\varvec{\sigma}} < {\varvec{\sigma}}_{s2}$, all components in parts 1, 2, and 3 in Fig. 2 participate in the creep work. The combined model is a five-element model (H–H/N-S/N), which can describe the creep characteristics of softer rocks. The corresponding equation of state can be expressed as

3 $$ \left\{ \begin{aligned} {\varvec{\sigma}}_{0} & = {\varvec{E}}_{0} {\varvec{\varepsilon}}_{0} \\ {\varvec{\sigma}}_{1} & = {\varvec{E}}_{1} {\varvec{\varepsilon}}_{1} + {\varvec{\eta}}_{1} \frac{{\user2{d\varepsilon }_{1} }}{{{\varvec{dt}}}} \\ {\varvec{\sigma}}_{2} & = {\varvec{\sigma}}_{s1} + {\varvec{\eta}}_{2} \frac{{\user2{d\varepsilon }_{2} }}{{{\varvec{dt}}}} \\ {\varvec{\sigma}} &= {\varvec{\sigma}}_{0} = {\varvec{\sigma}}_{1} = {\varvec{\sigma}}_{2} \\ {\varvec{\varepsilon}} &= {\varvec{\varepsilon}}_{0} + {\varvec{\varepsilon}}_{1} + {\varvec{\varepsilon}}_{2} \\ \end{aligned} \right. $$

According to Eq. (3), the one-dimensional creep equation of the five-element model is

4 $$ \varepsilon (t) = \frac{{\varvec{\sigma}}}{{{\varvec{E}}_{0} }} + \frac{\sigma }{{{\varvec{E}}_{1} }}\left( {1 - {\varvec{e}}^{{ - \frac{{{\varvec{E}}_{1} }}{{\eta_{1} }}t}} } \right) + \frac{{\sigma - \sigma_{s1} }}{{\eta_{2} }}{\varvec{t}} $$

When ${\varvec{\sigma}} \ge {\varvec{\sigma}}_{s1}$ and ${\varvec{\sigma}} \ge {\varvec{\sigma}}_{s2}$, all components in parts 1, 2, 3, and 4 in Fig. 2 participate in the creep work. The combined model is a seven-element model (H–H/N-S/N-S/N), which can describe the entire curve of soft rock creep. The state equation can be expressed as

5 $$ \left\{ \begin{aligned} {\varvec{\sigma}}_{0} &= {\varvec{E}}_{0} {\varvec{\varepsilon}}_{0} \\ {\varvec{\sigma}}_{1} &= {\varvec{E}}_{1} {\varvec{\varepsilon}}_{1} + {\varvec{\eta}}_{1} \frac{{\user2{d\varepsilon }_{1} }}{{{\varvec{dt}}}} \\ {\varvec{\sigma}}_{2} &= {\varvec{\sigma}}_{s1} + {\varvec{\eta}}_{2} \frac{{\user2{d\varepsilon }_{2} }}{{{\varvec{dt}}}} \\ {\varvec{\sigma}}_{3} &= {\varvec{\sigma}}_{s2} + {\varvec{\eta}}_{3} \frac{{\user2{d\varepsilon }_{{\varvec{3}}} }}{{{\varvec{dt}}}}\frac{1}{{\user2{\beta t}^{{{\varvec{\beta}} - 1}} }} \\ {\varvec{\sigma}} &= {\varvec{\sigma}}_{0} = {\varvec{\sigma}}_{1} = {\varvec{\sigma}}_{2} = {\varvec{\sigma}}_{3} \\ {\varvec{\varepsilon}} &= {\varvec{\varepsilon}}_{0} + {\varvec{\varepsilon}}_{1} + {\varvec{\varepsilon}}_{2} + {\varvec{\varepsilon}}_{3} \\ \end{aligned} \right. $$

According to Eq. (5), the one-dimensional constitutive equation of the seven-element model is

6 $$ {\varvec{\varepsilon}}({\varvec{t}}) = \frac{{\varvec{\sigma}}}{{{\varvec{E}}_{0} }} + \frac{{\varvec{\sigma}}}{{{\varvec{E}}_{1} }}\left( {1 - {\varvec{e}}^{{ - \frac{{{\varvec{E}}_{1} }}{{{\varvec{\eta}}_{1} }}t}} } \right) + \frac{{{\varvec{\sigma}} - {\varvec{\sigma}}_{s1} }}{{{\varvec{\eta}}_{2} }}{\varvec{t}} + \frac{{{\varvec{\sigma}} - {\varvec{\sigma}}_{{{\varvec{s}}2}} }}{{{\varvec{\eta}}_{3} }}t^{{\varvec{\beta}}} $$

### The three-dimensional form of creep of the viscoelastic-plastic model

Under the same stress condition, the compressive strength of rock samples obtained by creep test decreases, so the strength reduction caused by aging deformation of rock should be considered in the establishment of three-dimensional creep constitutive relationship. Therefore, the yield function and plastic potential function considering the aging deformation of rocks can be represented as $f^{\prime}$ and $g^{\prime}$, respectively. The determination method of their expressions is the same as that of the yield function $f$, which can be determined through creep tests^22^. The yield function expression is

7 $$ \begin{gathered} f\left( {\varvec{\sigma}} \right) = \sqrt {F\left[ {\left( {{\varvec{\sigma}}_{22} - {\varvec{\sigma}}_{33} } \right)^{2} + \left( {{\varvec{\sigma}}_{33} - {\varvec{\sigma}}_{11} } \right)^{2} } \right] + H\left( {{\varvec{\sigma}}_{11} - {\varvec{\sigma}}_{22} } \right)^{2} + 2\user2{L\tau }_{12}^{2} + 2M\left( {{\varvec{\tau}}_{31}^{2} + {\varvec{\tau}}_{32}^{2} } \right)} \hfill \\ \begin{array}{*{20}c} {} & {} & {} \\ \end{array}_{{}} + {\varvec{P}}\left( {{\varvec{\sigma}}_{11} + {\varvec{\sigma}}_{22} } \right) + \user2{Q\sigma }_{33} - 1 \hfill \\ \end{gathered} $$

For the associated flow rule, ${\varvec{g}} = f$; $\frac{{\partial {\varvec{g}}}}{{\partial {\varvec{\sigma}}}}$ is used to determine the direction of plastic strain. When the rock mass reaches the yield condition, further loading will cause plastic flow. The flow law can be used to determine or assume the direction of plastic flow.

The rock mass is in a complex three-dimensional stress state in actual engineering. The stress tensor can be decomposed into spherical stress tensor ${\varvec{\sigma}}_{m}$ and deviatoric stress tensor ${\varvec{S}}_{{{\varvec{ij}}}}$, with their expressions as follows,

8 $$ {\varvec{\sigma}}_{{\varvec{m}}} = \frac{1}{3}\left( {\sigma_{11} + \sigma_{22} + \sigma_{33} } \right) $$

9 $$ {\varvec{S}}_{{{\varvec{ij}}}} = {\varvec{\sigma}}_{{{\varvec{ij}}}} - {\varvec{\delta}}_{{{\varvec{ij}}}} \sigma_{{\varvec{m}}} $$

Generally, the spherical stress tensor ${\varvec{\sigma}}_{m}$ can cause a change in the volume of rock mass, but it cannot change its shape. The deviatoric stress tensor ${\varvec{S}}_{{{\varvec{ij}}}}$ can only cause a change in the shape but cannot change its volume. Correspondingly, the strain tensor of the rock mass can be decomposed into spherical strain tensor ${\varvec{\varepsilon}}_{{\varvec{m}}}$ and partial strain tensor ${\varvec{\varepsilon}}_{{{\varvec{ij}}}}$. The corresponding expression can be written as

10 $$ \varepsilon_{{\varvec{m}}} = \frac{1}{3}\left( {\varepsilon_{11} + \varepsilon_{22} + \varepsilon_{33} } \right) $$

11 $$ {\varvec{e}}_{{{\varvec{ij}}}} = \varepsilon_{{{\varvec{ij}}}} - \delta_{{{\varvec{ij}}}} \varepsilon_{{\varvec{m}}} $$

The elastic modulus *E* and Poisson’s ratio ${\varvec{\upsilon}}$ of the rock mass in the one-dimensional creep equation should be corresponding to the bulk modulus *G* and shear modulus *K* in the three-dimensional creep equation, which can be expressed as

12 $$ {\varvec{G}} = \frac{{\varvec{E}}}{{2(1 + {\varvec{\upsilon}})}} $$

13 $$ {\varvec{K}} = \frac{{\varvec{E}}}{{3(1 - 2{\varvec{\upsilon}})}} $$

According to Eqs. (1)–(13), combined with the principle of superposition, the creep equation under the three-dimensional stress state of the rock mass can be obtained,

14 $$ {\varvec{e}}_{{{\varvec{ij}}}} (t) = \frac{{{\varvec{\delta}}_{{{\varvec{ij}}}} {\varvec{\sigma}}_{m} }}{{3{\varvec{K}}_{0} }} + \frac{{{\varvec{S}}_{{{\varvec{ij}}}} }}{{2{\varvec{G}}_{1} }}\left( {1 - {\varvec{e}}^{{ - \frac{{{\varvec{G}}_{1} }}{{{\varvec{\eta}}_{1} }}{\varvec{t}}}} } \right) + \frac{{{\varvec{H}}_{1} ({\varvec{F}}_{1} )}}{{{\varvec{\eta}}_{2} }}\frac{{\partial {\varvec{Q}}_{1} }}{{\partial {\varvec{\sigma}}_{{{\varvec{ij}}}} }}t + \frac{{{\varvec{H}}_{2} ({\varvec{F}}_{2} )}}{{{\varvec{\eta}}_{3} }}\frac{{\partial {\varvec{Q}}_{2} }}{{\partial {\varvec{\sigma}}_{{{\varvec{ij}}}} }}{\varvec{t}}^{{\varvec{\beta}}} $$

where ${\varvec{\sigma}}_{s1}$ and ${\varvec{\sigma}}_{s2}$ are the yield strength of the rock mass in steady state creep and accelerated creep of the rock mass, respectively. *β* is the component parameter of the nonlinear Newtonian body, which is determined by the test. ${\varvec{H}}\left( {\varvec{\sigma}} \right)$ is the unit step function, ${\varvec{F}}$ is the rock mass yield function^23^, ${\varvec{Q}}$ is the plastic potential function, and adopts the associated flow rule, which are expressed as

15 $$ {\varvec{H}}({\varvec{\sigma}}) = \left\{ \begin{gathered} 0,{\varvec{\sigma}} < 0 \hfill \\ {\varvec{\sigma}},{\varvec{\sigma}} \ge 0 \hfill \\ \end{gathered} \right. $$

16 $$ {\varvec{F}} = \sqrt {{\varvec{J}}_{2} } - \frac{{{\varvec{\sigma}}_{s} }}{\sqrt 3 } $$

17 $$ {\varvec{Q}} = {\varvec{F}} $$

In Eq. (16), $J_{2}$ is the second deviator stress invariant.

## Development of numerical program for viscoelastic-plastic model

### Finite element analysis method of viscoelastic-plastic model

In the calculation process of the finite element method, according to the one-dimensional and three-dimensional viscoelastic-plastic creep equations given above, it needs to be expressed as the corresponding incremental equation form. The total strain increment $\Delta {\varvec{\varepsilon}}$ includes three parts: elastic strain increment $\Delta {\varvec{\varepsilon}}_{{\varvec{e}}}$, viscoelastic strain increment $\Delta {\varvec{\varepsilon}}_{{{\varvec{ev}}}}$ and viscoplastic strain increment $\Delta {\varvec{\varepsilon}}_{{{\varvec{vp}}}}$.

18 $$ \left[ {\Delta {\varvec{\varepsilon}}} \right]\user2{ = }\left[ {\Delta {\varvec{\varepsilon}}_{{\varvec{e}}} } \right]\user2{ + }\left[ {\Delta {\varvec{\varepsilon}}_{{{\varvec{ev}}}} } \right]\user2{ + }\left[ {\Delta {\varvec{\varepsilon}}_{{{\varvec{vp}}}} } \right] $$

The incremental form of the elastic element in part 1 in Fig. 2 is given by

19 $$ \left[ {\Delta {\varvec{\varepsilon}}_{{\varvec{e}}} } \right] = \left[ {{\varvec{D}}^{ - 1} } \right]\left[ {\Delta {\varvec{\sigma}}} \right] $$

The incremental form of the viscoelastic element in part 2 in Fig. 2 is given by

20 $$ \left[ {\Delta {\varvec{\varepsilon}}_{{{\varvec{ve}}}} } \right]_{{\user2{t + \Delta t}}} = \left[ {\Delta {\varvec{\varepsilon}}_{{\varvec{e}}} } \right]_{{\varvec{t}}} \cdot {\varvec{e}}^{{ - \frac{{{\varvec{G}}_{1} }}{{{\varvec{\eta}}_{1} }}\Delta t}} + \frac{{{\varvec{G}}_{0} }}{{{\varvec{G}}_{1} }}\left[ {\varvec{D}} \right]^{ - 1} \left[ {\varvec{\sigma}} \right] \cdot \left( {1 - {\varvec{e}}^{{ - \frac{{{\varvec{G}}_{1} }}{{{\varvec{\eta}}_{1} }}\Delta {\varvec{t}}}} } \right) $$

21 $$ \left[ {\Delta {\varvec{\varepsilon}}_{{{\varvec{ve}}}} } \right] = \left[ {\Delta {\varvec{\varepsilon}}_{{{\varvec{ve}}}} } \right]_{{\user2{t + \Delta t}}} - \left[ {\Delta {\varvec{\varepsilon}}_{{{\varvec{ve}}}} } \right]_{{\varvec{t}}} $$

The incremental form of the viscoplastic element in part 3 in Fig. 2 is given by

22 $$ \left[ {\dot{\user2{\varepsilon }}_{{{\varvec{vp}}}} } \right]_{{\varvec{t}}} = \frac{{{\varvec{H}}\left[ {{\varvec{F}}_{1} } \right]}}{{{\varvec{\eta}}_{2} }}\frac{{\partial {\varvec{Q}}_{1} }}{{\partial {\varvec{\sigma}}_{ij} }} $$

The incremental form of the viscoplastic element in part 4 in Fig. 2 is given by

23 $$ \left[ {\dot{\user2{\varepsilon }}_{{{\varvec{vp}}}} } \right]_{{\varvec{t}}} = {\varvec{\beta}}\frac{{{\varvec{H}}\left[ {{\varvec{F}}_{2} } \right]}}{{{\varvec{\eta}}_{3} }}\frac{{\partial {\varvec{Q}}_{2} }}{{\partial {\varvec{\sigma}}_{ij} }}{\varvec{t}}^{{{\varvec{\beta}} - 1}} $$

Then the sum of the viscoplastic strain increments of parts 3 and 4 can be expressed as

24 $$ \left[ {\Delta {\varvec{\varepsilon}}_{{{\varvec{vp}}}} } \right] = \left( {\frac{{{\varvec{H}}\left[ {{\varvec{F}}_{1} } \right]}}{{{\varvec{\eta}}_{2} }}\frac{{\partial {\varvec{Q}}_{1} }}{{\partial {\varvec{\sigma}}_{ij} }} + {\varvec{\beta}}\frac{{{\varvec{H}}\left[ {{\varvec{F}}_{2} } \right]}}{{{\varvec{\eta}}_{3} }}\frac{{\partial {\varvec{Q}}_{2} }}{{\partial {\varvec{\sigma}}_{ij} }}{\varvec{t}}^{{{\varvec{\beta}} - 1}} } \right)\Delta {\varvec{t}} $$

Denote the creep increment as $\Delta {\varvec{\varepsilon}}_{{\varvec{c}}}$, then

25 $$ \left[ {\Delta {\varvec{\varepsilon}}_{{\varvec{c}}} } \right] = \left[ {\Delta {\varvec{\varepsilon}}_{{{\varvec{ve}}}} } \right] + \left[ {\Delta {\varvec{\varepsilon}}_{{{\varvec{vp}}}} } \right] $$

26 $$ \left[ {\Delta {\varvec{\varepsilon}}_{{\varvec{e}}} } \right] = \left[ {\Delta {\varvec{\varepsilon}}} \right] - \left[ {\Delta {\varvec{\varepsilon}}_{{\varvec{c}}} } \right] $$

27 $$ \left[ {\Delta {\varvec{\sigma}}} \right] = \left[ {\varvec{D}} \right]\left[ {\Delta {\varvec{\varepsilon}}_{{\varvec{e}}} } \right] $$

where $\left[ {\varvec{D}} \right]$ is the elastic stiffness matrix, $\left[ {\Delta {\varvec{\sigma}}} \right]$ is the stress increment matrix, $\left[ {\Delta {\varvec{\varepsilon}}} \right]$ is the strain increment matrix.

### Development of subroutine for viscoelastic-plastic model

ABAQUS finite element software provides users with a secondary development interface for subprograms. Users can write programs in Fortran and define material subprograms (UMAT). It realizes data exchange with the main program through the interface of the Standard solver. According to the ABAQUS nonlinear incremental loading and Newton equilibrium iteration algorithm (incremental iteration method), the software calls UMAT for each calculation unit at each incremental step to obtain the Jacobian matrix DDSDDE (stiffness coefficient matrix $\left[ {\varvec{D}} \right]$). Then, it is passed to the ABAQUS main program through the Standard interface, where the balance check is completed. If the balance check does not meet the error requirements, ABAQUS will continue to iterate until the error requirements are met before proceeding to the next incremental step of solution. It can be seen that UMAT is called frequently, so the quality of the subprogram code should be fully considered to improve the computational efficiency.

The viscoelastic-plastic model proposed in this paper is used to solve time-related nonlinear problems. Its stiffness matrix changes with time. Each incremental calling UMAT requires recalculating the stiffness matrix, greatly reducing computational efficiency. Hence, this paper uses the constant stiffness method for calculation, and updates the stress increment $\Delta {\varvec{\varepsilon}}_{{{\varvec{n}} + 1}}$ and state variables $\Delta {\varvec{\sigma}}_{{{\varvec{n}} + 1}}$ of the subroutine through the strain increment within the time increment $\Delta {\varvec{t}}_{{\varvec{n}}} = {\varvec{t}}_{{{\varvec{n}} + 1}} - {\varvec{t}}_{{\varvec{n}}}$. According to Eqs. (19)–(27), the following can be derived,

28 $$ \left[ {\Delta {\varvec{\sigma}}} \right] = \left[ {\varvec{D}} \right]\left\{ {\left[ {\Delta {\varvec{\varepsilon}}} \right] - \left[ {\Delta {\varvec{\varepsilon}}_{{\varvec{c}}} } \right]} \right\} $$

29 $$ \left[ {\varvec{\sigma}} \right]_{{{\varvec{n}} + 1}} = \left[ {\varvec{\sigma}} \right]_{{\varvec{n}}} + \left[ {\Delta {\varvec{\sigma}}} \right] $$

30 $$ \left[ {\dot{\user2{\varepsilon }}_{{\varvec{c}}} } \right]_{{\varvec{n}}} = \frac{{\left[ {\Delta {\varvec{\varepsilon}}_{{\varvec{c}}} } \right]_{{\varvec{n}}} }}{{\Delta {\varvec{t}}_{{\varvec{n}}} }} $$

In order to improve the calculation accuracy, $\left[ {\Delta \varepsilon_{c} } \right]_{n}$ is calculated using the following difference method.

31 $$ \left[ {\Delta {\varvec{\varepsilon}}_{{\varvec{c}}} } \right]_{{\varvec{n}}} = \Delta {\varvec{t}}_{{\varvec{n}}} \left\{ {\left( {1 - \theta } \right)\left[ {\dot{\user2{\varepsilon }}_{{\varvec{c}}} } \right]_{n} + {\varvec{\theta}}\left[ {\dot{\user2{\varepsilon }}_{{\varvec{c}}} } \right]_{{{\varvec{n}} + 1}} } \right\} $$

32 $$ \left[ {\dot{\user2{\varepsilon }}_{{\varvec{c}}} } \right]_{{{\varvec{n}} + 1}} = \left[ {\dot{\user2{\varepsilon }}_{{\varvec{c}}} } \right]_{{\varvec{n}}} + \left( {\frac{{\partial \dot{\user2{\varepsilon }}_{{\varvec{c}}} }}{{\partial {\varvec{\sigma}}}}} \right)_{{\varvec{n}}} \left[ {\Delta {\varvec{\sigma}}_{{\varvec{n}}} } \right], $$

where *n* is an incremental step, and ${\varvec{\theta}}$ is an integral parameter between 0 and 1^24^. ${\varvec{\theta}} = 0$ corresponds to an explicit Euler integration algorithm, and ${\varvec{\theta}} = 1$ corresponds to an implicit Euler integration algorithm.

**Figure 3:**
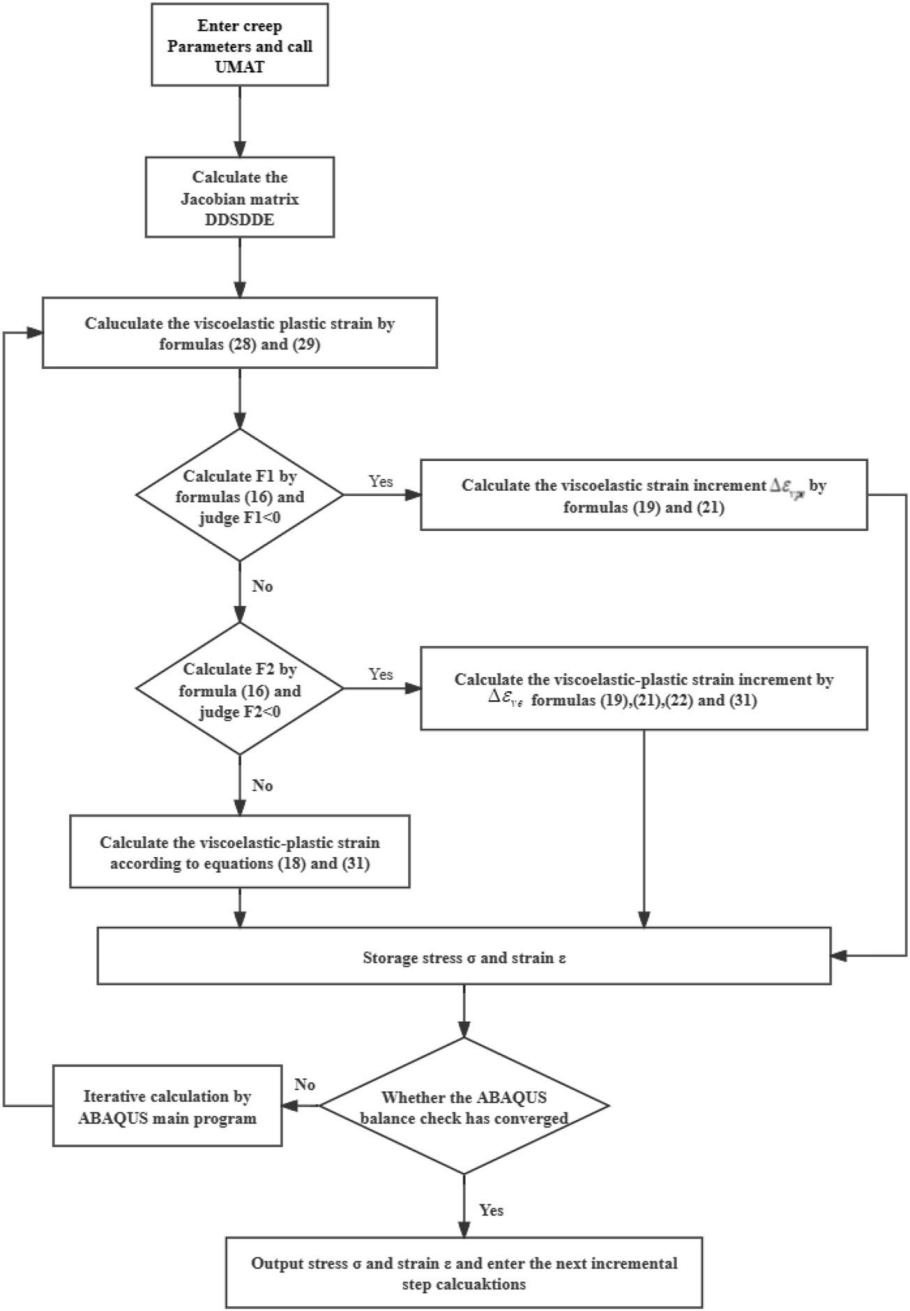
Flow chart of UMAT of visco-elastoplastic model.

The viscoelastic-plastic constitutive model is implemented in ABAQUS through UMAT. At the beginning of each increment step, the ABAQUS main program passes the strain increment and time increment step at the integral point of the unit, as well as the current known state of stress, strain and other variables related to the solution process. UMAT calculates the stress increment and updates the stress and state variables according to the constitutive equation, and provides the Jacobian matrix DDSDDE to the ABAQUS main program to form the overall stiffness matrix. The main program solves the displacement increment according to the current load increment, and then complete the balance check. If the balance check does not meet the error requirements, ABAQUS will iterate until convergence, and then proceed to the next incremental step. In summary, the main UMAT process of the model is shown in Fig. 3.

### Example verification of viscoelastic plastic UMAT

**Figure 4:**
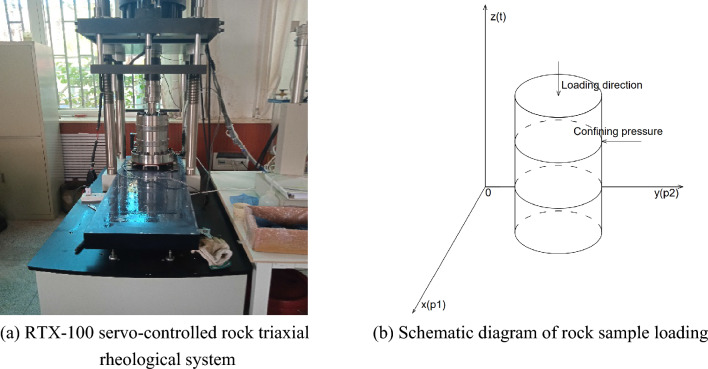
Test instruments and schematic diagram of rock sample loading.

In order to verify the correctness and rationality of the viscoelastic-plastic seven-element model proposed in this paper, the RTX-1000 servo-controlled rock triaxial rheological system of Chang'an University is adopted to conduct triaxial rheological tests on soft and hard rock samples (bedding inclination angle *θ* = 0°) under a confining pressure of 15 MPa and a uniformly distributed load of 100 MPa, as shown in Fig. 4. During the test, the confining pressure is first loaded to the specified value, and then the axial pressure is applied. The axial strain of the sample is recorded throughout the test, and the strain recording interval is 15 s in the loading stage and 60 s in the creep stage. The time-strain curve is drawn after the test, and the test results are shown in Fig. 6.

**Figure 5:**
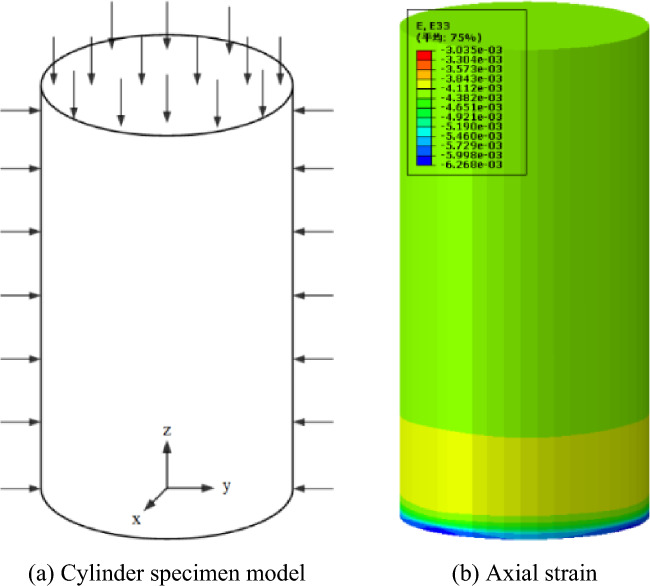
Numerical analysis model of triaxial compression creep test.

At the same time, a cylindrical specimen model with a diameter of 35.5 mm and a height of 70 mm is established, consistent with the triaxial rheological test conditions. The bottom of the cylindrical specimen is fixed ($U_{x} = U_{y} = U_{z} = 0$), a uniformly distributed load of 100 MPa is applied to the top of the specimen, and a confining pressure of 15 MPa is applied around the specimen, as shown in Fig. 5(a). Through the viscoelastic-plastic UMAT developed in the paper, the numerical calculation is completed in ABAQUS. The axial strain cloud diagram is shown in Fig. 5(b). The rheological and mechanical parameters of the rock used are shown in Table 1.

**Table 1:** Comparison of the creep parameters between Hohai model and the present model^25,26^.

Creep model	${\varvec{E}}_{0} /GPa$	${\varvec{E}}_{1} /GPa$	${\varvec{E}}_{2} /GPa$	${\varvec{\eta}}_{1} /\left( {GPa \cdot h} \right)$	${\varvec{\eta}}_{2} /\left( {GPa \cdot h} \right)$	${\varvec{\eta}}_{3} /\left( {GPa \cdot h} \right)$	${\varvec{\beta}}$	${\varvec{n}}$
HoHai model	25.8	715.30	96.9	62.40	792	49,927.2	–	12.673
Paper model	25.8	166.67	–	49.38	728	49,999.2	8.65	–

*n* is the rheological index, reflecting the speed of the accelerated rheological rate.

**Figure 6:**
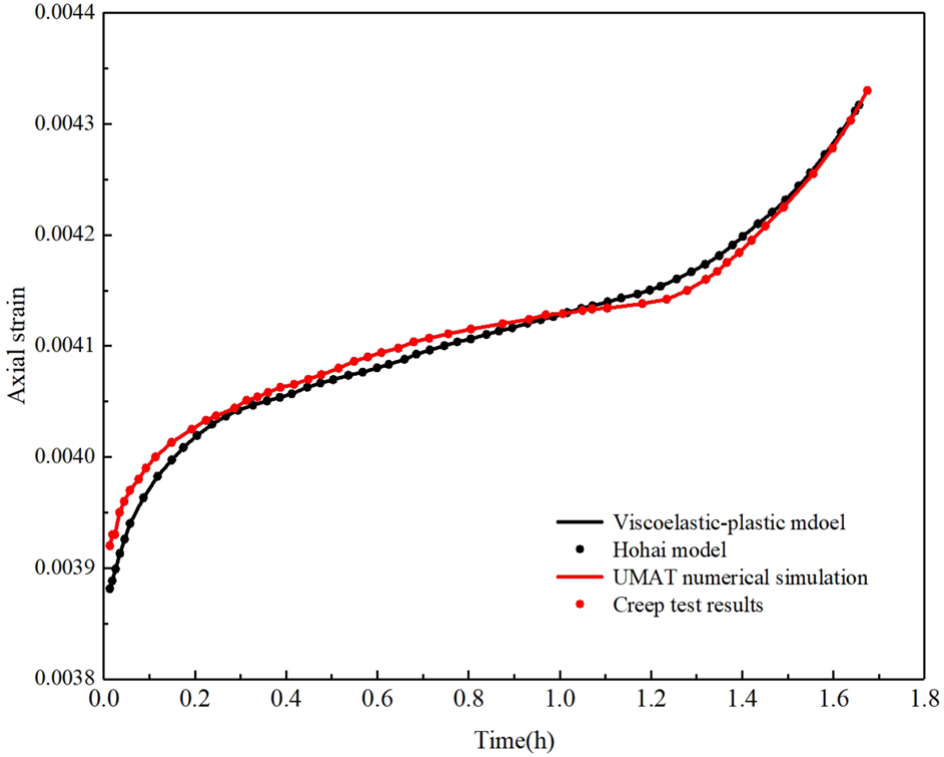
Comparison of numerical analysis results of Hohai model and Viscoelastic-plastic model by UMAT.

Figure 6 shows the analytical solution of the viscoelastic-plastic seven-element model and the numerical calculation result of the UMAT subroutine for the axial strain creep curve of the cylindrical specimen, and compares them with the analytical solution of the HoHai model and the creep test results. It can be found that in the stages of attenuation creep and steady-state creep, the calculation results of the UMAT subroutine based on the viscoelastic-plastic model are better than the analytical solutions of the HoHai model in fitting the creep test results. In the accelerated creep stage, the creep curves of the four are highly consistent, thus verifying the rationality of the viscoelastic plastic model and the effectiveness of the UMAT subroutine.

## Numerical simulation of tunnel excavation and its calculation results

### Numerical model of tunnel excavation

**Figure 7:**
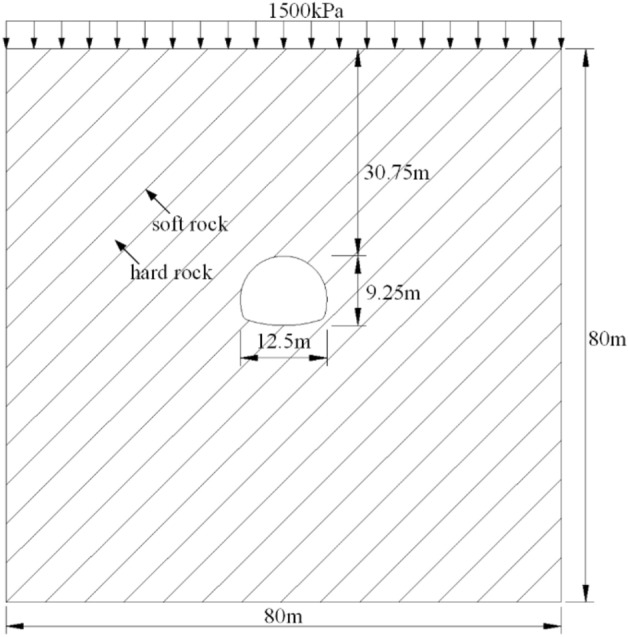
Excavation model of interbedded rock mass tunnel.

**Figure 8:**
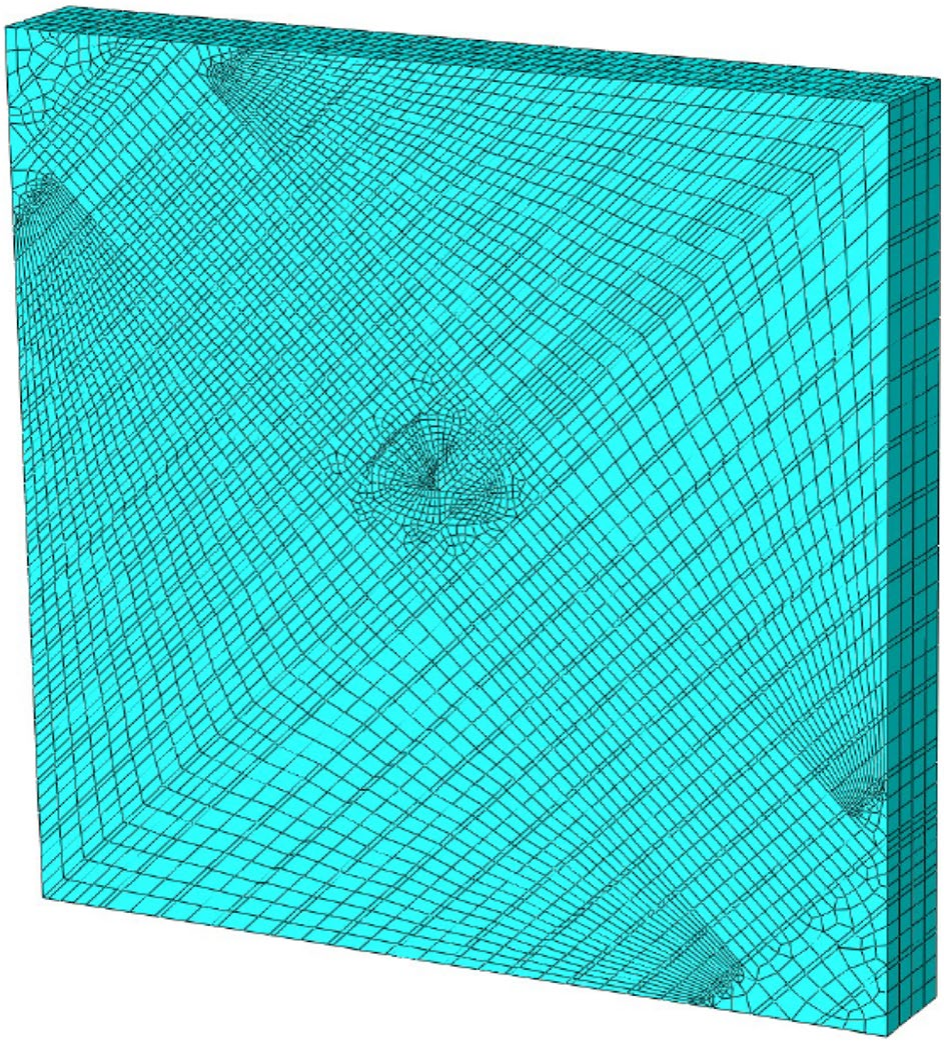
Mesh part model.

On the basis of the UMAT subroutine of the viscoelastic-plastic model developed in this paper, the geometric model shown in Figs. 7 and 8 is established based on the ABAQUS finite element software to study the creep behavior of tunnels in soft and hard interbedded rock masses. The numerical model of tunnel excavation is established in an 80 $\times$ 80 rectangular interbedded rock mass area. The bottom of the tunnel is 40 m away from the horizontal ground. The net width of the tunnel is 12.5 m, and the net height of the tunnel is 9.25 m. A uniform pressure of p = 1500 kPa (rock weight $\gamma = 25kN/m^{3}$) is applied to the ground to simulate the ground stress at a depth of 60 m, that is, the depth of the tunnel is about 90.75 m. Constraints are set at the boundary of the rectangular area to limit the horizontal displacement of the left and right boundary of the model and the horizontal and vertical displacement of the bottom of the model. According to Eq. (33), the ground stress of the model is balanced and the initial ground stress value is set.

33 $$ \left\{ \begin{gathered} \sigma_{v} = p + \gamma h \hfill \\ \sigma_{h} = \lambda \sigma_{v} \hfill \\ \lambda = \frac{\nu }{1 - \nu } \hfill \\ \end{gathered} \right. $$

where $\sigma_{v}$ and $\sigma_{h}$ are the lateral and vertical stress (MPa), $p$ is the upper load pressure (MPa). $\gamma$ is the natural bulk weight (KN/m^3^). *h* is the depth of the unit body to the surface. $\lambda$ is the side pressure coefficients, $\nu$ is Poisson coefficient.

In the process of establishing the numerical model, the rock mass is divided into soft rock and hard rock regions according to the rock inclination. Soft rock and hard rock are distributed alternately (hard rock thickness is 3.6 m, soft rock thickness is 0.4 m). Different creep mechanical parameters are set, and the parameters are shown in Table 2. The numerical model is used to simulate the full section excavation process of the tunnel and the creep behavior of rock mass within 24 h after excavation. Due to the time effect after tunnel excavation, the ground displacement will extend from the surface direction of the tunnel. The corresponding ground displacement is mainly concentrated in the ground settlement and horizontal displacement, and convergence around the tunnel. Therefore, the numerical simulation study considers the influence of tunnel excavation in interbedded rock mass with different rock inclination angles on ground horizontal displacement, ground settlement and convergence around the tunnel. The specific simulation test scheme is shown in Table 3.

**Table 2:** Creep test parameters of soft rock and hard rock^16^.

Lithology	${\varvec{E}}_{0} /GPa$	*μ*	${\varvec{E}}_{1} /GPa$	${\varvec{\eta}}_{1} /\left( {GPa \cdot h} \right)$	${\varvec{\eta}}_{2} /\left( {GPa \cdot h} \right)$	${\varvec{\eta}}_{3} /\left( {GPa \cdot h} \right)$	_*Rf*_	_*t*/s_
Carbonaceous marl	2.013	0.3	54.39	150.8	7105	10,000	3	10
Shale	0.348	0.3	39.14	52.9	967.6	10,000	3	10

**Table 3:** Simulation test plan for tunnel excavation.

Influencing factors	Numerical simulation plan
Rock inclination $\left( {\varvec{\alpha}} \right)$	0°, 15°, 30°, 45°, 60°, 75°, 90°

### Numerical calculation results of tunnel excavation

#### The influence of different rock inclinations on the horizontal displacement of the ground

**Figure 9:**
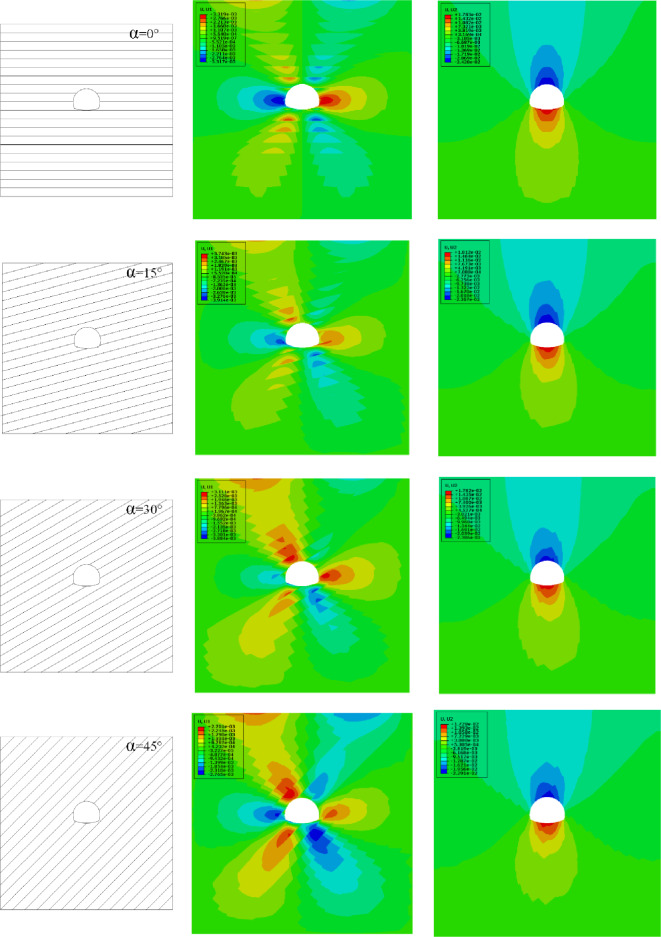

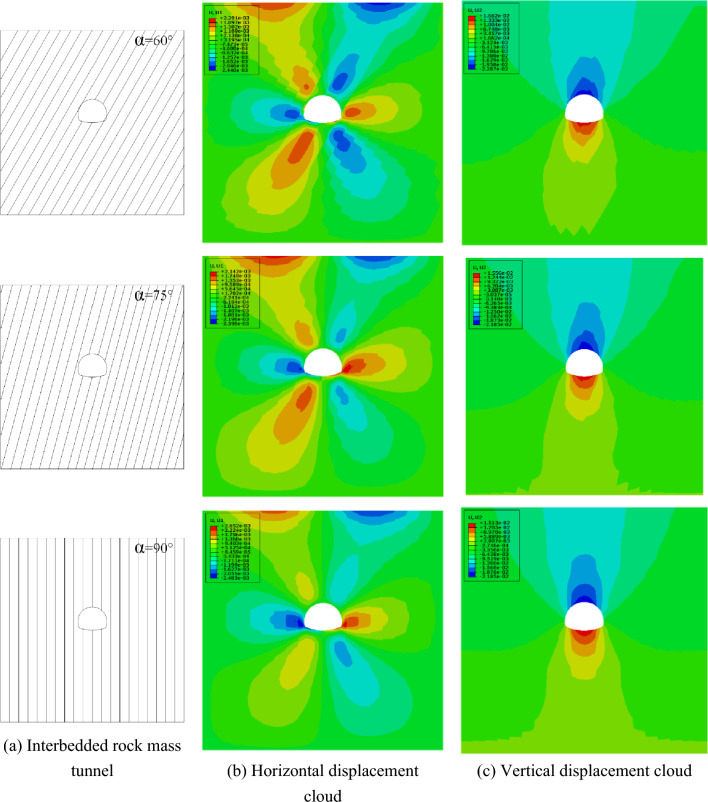
Numerical calculation model of different rock inclination angle.

**Figure 10:**
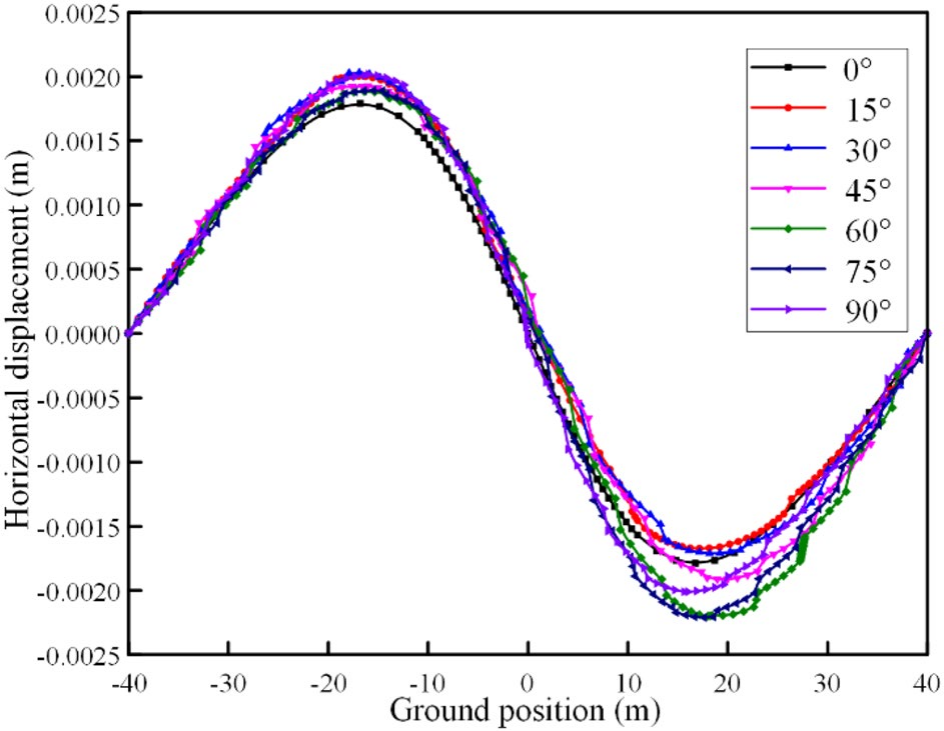
Ground horizontal displacement.

In order to study the influence of tunnel excavation in interbedded rock mass on the ground horizontal displacement within 80 m above the tunnel when considering time-dependent deformation, numerical simulations are carried out for rock mass with different rock inclination angles (${\varvec{\alpha}}$ = 0°, 15°, 30°, 45°, 60°, 75°, 90°). The horizontal displacement cloud map is shown in Fig. 9b. It can be found that the maximum ground horizontal displacement occurs between 15–20 m on both sides of the ground directly above the tunnel. It is specified that the horizontal displacement is positive to the right. The numerical calculation results are shown in Fig. 10.

**Figure 11:**
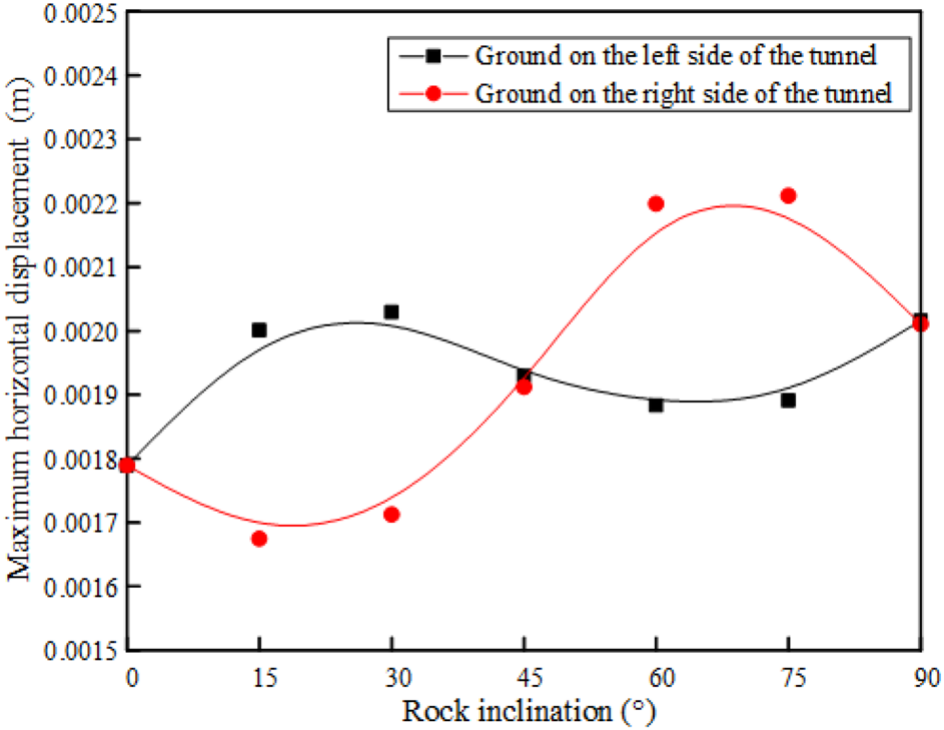
Maximum horizontal displacement of the ground at different rock inclinations.

After the tunnel is excavated in the rock mass with different rock inclination angles, there is a significant difference in the maximum horizontal displacement of the ground on the left and right sides of the tunnel, as shown in Fig. 11. It can be found that with the continuous increase of the rock inclination, the maximum horizontal displacement of the ground on the left side of the tunnel shows a "N" trend. When the rock inclination is 0°, the horizontal displacement of the ground on the left of the tunnel reaches the minimum value. When the rock inclination is 30° or 90°, the horizontal displacement of the ground on the left side of the tunnel reaches its maximum value. However, the maximum horizontal displacement of the ground on the right side of the tunnel shows the opposite trend with the change of the inclination on the left side of the tunnel. When the inclination angle of the rock layer is 15° and 75°, the horizontal displacement of the right ground reaches the minimum and the maximum value, respectively. Affected by the inclination of the rock formation, the rock and soil slipped along the interlayer under the action of self-weight stress after tunnel excavation. Therefore, the horizontal displacement of the ground on the right side of the tunnel is greater than that on the left side of the tunnel.

In addition, when the rock layer inclination angle is 0°, 45° and 90°, the horizontal displacement of the left and right vertical ground of the tunnel is basically the same. When 0° < ${\varvec{\alpha}}$ < 45°, the horizontal displacement of the ground on the left side above the tunnel is more obvious. When 45° < ${\varvec{\alpha}}$ < 90°, the horizontal displacement of the ground on the right side above the tunnel is more obvious. The left and right vertical asymmetric horizontal displacement above the tunnel caused by the change of the rock layer inclination is a local prominent problem in the tunnel excavation process, which puts forward higher requirements for the control of ground deformation in engineering practice.

#### The influence of rock inclination on the ground settlement

**Figure 12:**
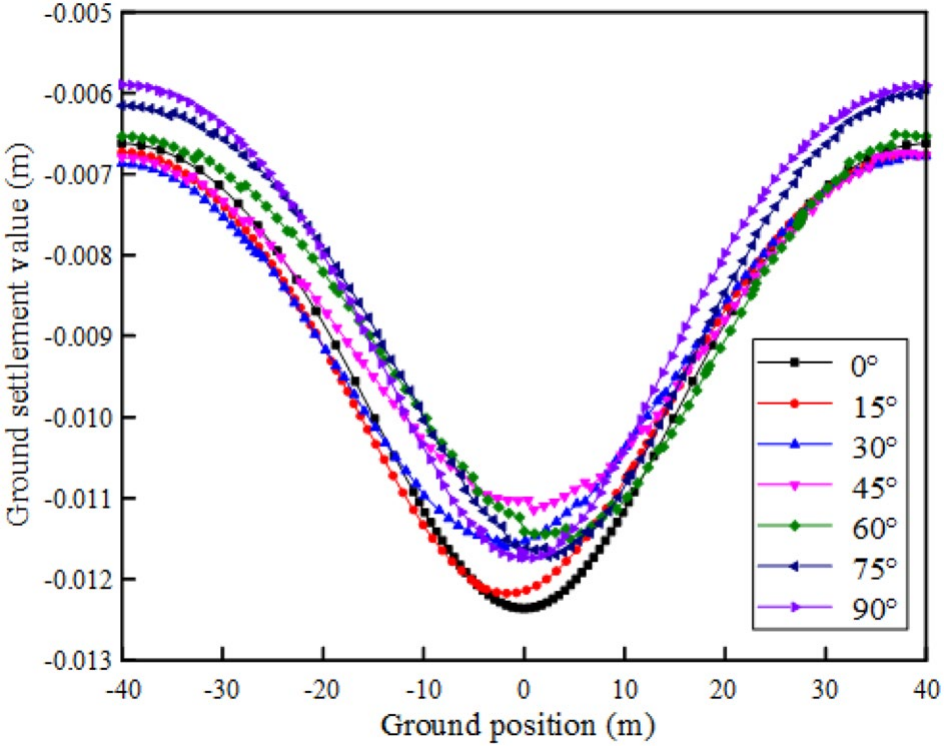
Ground settlement.

In order to study the influence of interbedded rock mass tunnel excavation on the ground settlement within 80 m above the tunnel when considering the effect of aging deformation, numerical simulations on rock masses with different rock inclination angles (${\varvec{\alpha}}$ = 0°, 15°, 30°, 45°, 60°, 75°, 90°) are carried out. The settlement deformation displacement cloud map is shown in Fig. 9c. It can be found that the maximum ground settlement occurs within 5 m of the ground on the left and right sides of the tunnel. Extending along the ground on both sides of the tunnel, the ground settlement gradually decreases, and the vertical displacement upward is defined as positive. The numerical calculation results are shown in Fig. 12.

**Figure 13:**
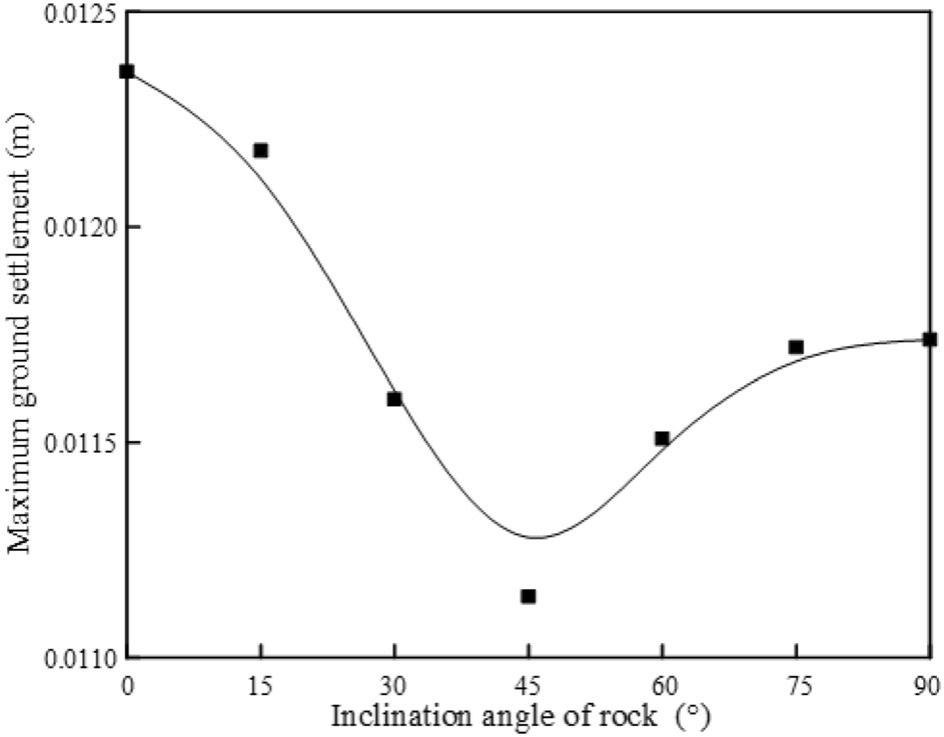
Maximum ground settlement of different inclination.

When tunnels are excavated in rock masses with different rock inclination angles, the maximum ground settlement is obviously different, as shown in Fig. 13. With the change of rock layer inclination from 0° to 90°, the maximum ground settlement decreases rapidly at first, and then increases slowly with the increase of rock layer inclination. When the inclination angle of the rock layer is greater than 75°, its value no longer increases, and the maximum settlement changes in a "V" shape. The maximum and minimum settlement are obtained when the rock inclination is 0° and 45°, respectively.

Numerical analysis results show that tunnel excavation in a rock mass with a rock inclination angle of 45° has lower requirements for ground settlement deformation control. And when α = 0°, the problem of ground settlement caused by tunnel excavation is more prominent.

#### The influence of the rock inclination on the convergence of the tunnel periphery

**Figure 14:**
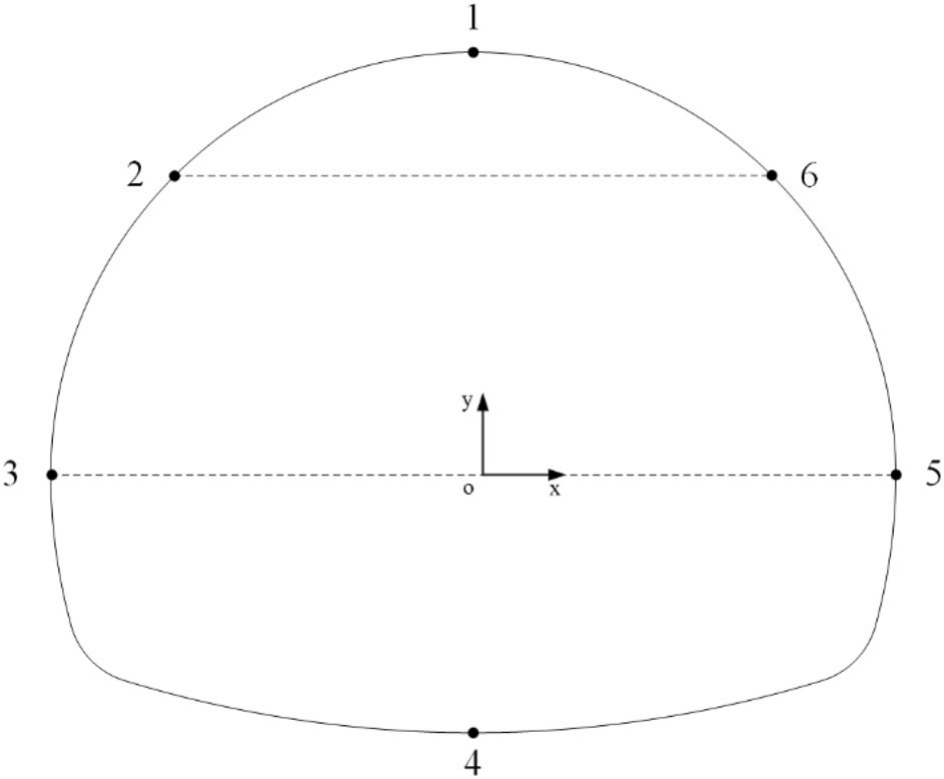
Layout of observation points of tunnel section.

In order to better study the influence of the tunnel excavation of interbedded rock mass on the convergence of the tunnel periphery when considering time-dependent deformation, this paper conducted numerical simulations on the rock masses with different rock inclination angles(${\varvec{\alpha}}$ = 0°, 15°, 30°, 45°, 60°, 75°, 90°). The displacement cloud map is shown in Fig. 9. Six observation points (1-vault, 2-left arched shoulder, 3-left arch waist, 4-arch bottom, 5-right arch waist and 6-right arched shoulder) are selected along the tunnel excavation outline, as shown in Fig. 14. The absolute value of the vertical displacement at observation points 1 and 4 is taken as the convergence value. The absolute value of the relative horizontal displacement of the left and right arch shoulder observation points 2 and 6 ($U_{26} = \left| {U_{x2} - U_{x6} } \right|$) is taken as the arch shoulder convergence value. The absolute value of the relative horizontal displacement of the left and right arch waist observation points 3 and 5($U_{35} = \left| {U_{x3} - U_{x5} } \right|$) is taken as the arch waist convergence value. The absolute value of the settlement amount of vault observation point 1 and the rebound amount of the arch bottom observation point 4 ($\left| {U_{y1} } \right|$ and $\left| {U_{y4} } \right|$) is taken as the convergence value of vault and arch bottom, respectively.

**Figure 15:**
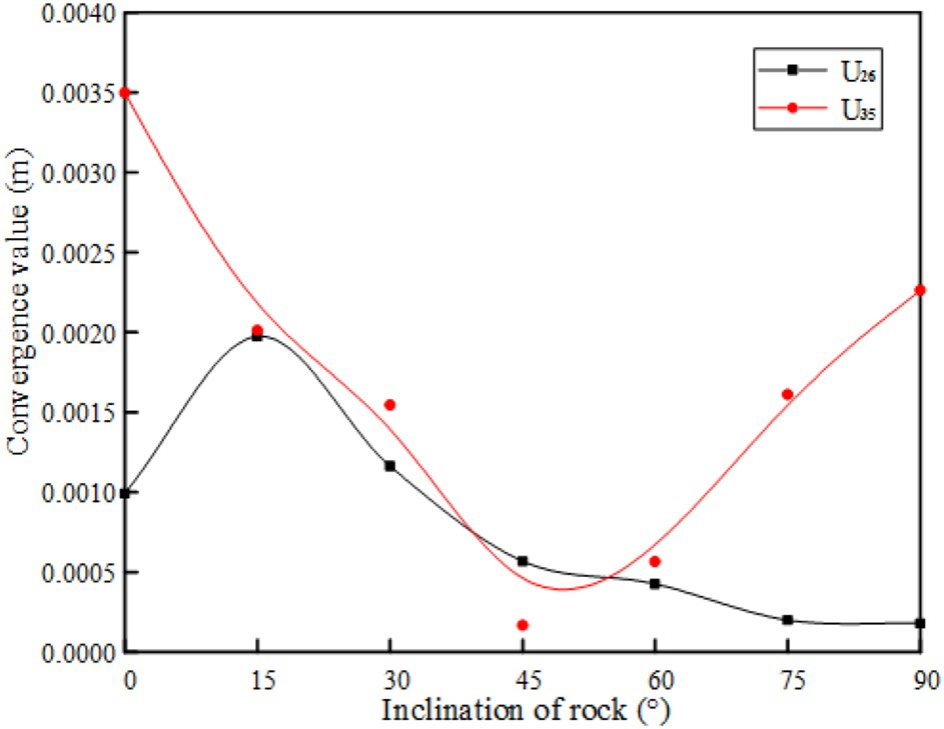
Convergence value of spandrel and arch waist of rock with different inclination.

**Figure 16:**
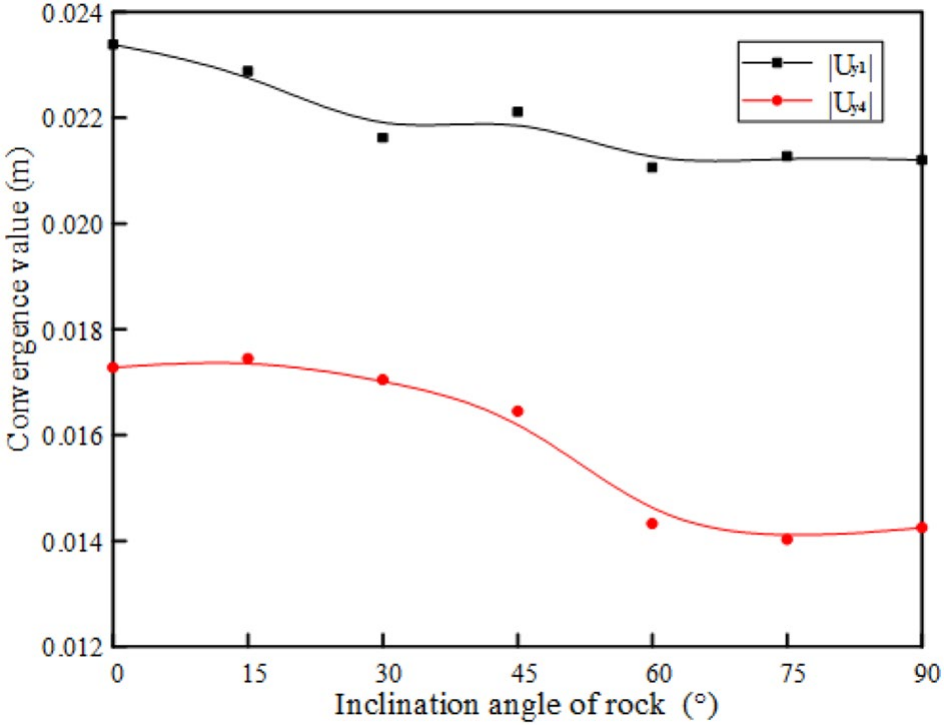
Convergence values of vault and arch bottom of rock with different inclination.

The numerical calculation results obtained according to the simulation scheme are shown in Figs. 15 and 16. The convergence values of the arch shoulder and arch waist of the tunnel are shown in Fig. 15. It is found that the convergence value of the arch shoulder increases rapidly with the increase of the rock inclination, and then slowly decreases. When ${\varvec{\alpha}}$ is 15° and 90°, the convergence value of the arch shoulder is the largest and the minimum, respectively. However, the change trend of the arch waist convergence value is opposite, which first decreases and then increases with the increase of the rock inclination angle ${\varvec{\alpha}}$. When ${\varvec{\alpha}}$ is 0° and 45°, the convergence value of the arch waist is the largest and the minimum, respectively. In addition, when 15° < ${\varvec{\alpha}}$ < 60°, the convergence value of the arch shoulder is close to the arch waist, but the convergence value of the arch waist is generally greater than that of the arch shoulder, that is, the deformation of the arch waist is greater than that of the arch shoulder. As shown in Fig. 16, the settlement of the arch top and the rebound of the arch bottom are consistent with the variation trend of the inclination angle of the rock layer. The convergence value gradually decreases with the increase of the inclination angle of the rock layer, and the maximum and minimum values are obtained when $\alpha$ is close to 0° and 90°, respectively.

The results of the convergence calculations around the tunnel show that the maximum deformation of the surrounding rock of the tunnel is affected by the inclination of the rock formation. The maximum deformation is also the dangerous part of the tunnel excavation, as shown in Fig. 9b. When the rock inclination is close to 0° or 90°, the dangerous part of the tunnel section appears at the arch waist. When 15°$\le {\varvec{\alpha}} \le^{ \circ }$ 75°, the dangerous part appears at the arch shoulder.

## Conclusion

1. Based on the rheological characteristics of soft and hard cross-layered rock mass, a viscoelastic-plastic seven-element combination model considering the stress threshold is proposed. The creep equation of the model in the three-dimensional stress state is derived. At the same time, combined with the finite element analysis method, the secondary development of the UMAT subroutine is completed in ABAQUS software. The rationality of the model and the effectiveness of the UMAT subprogram are verified by comparing the analytical solution of the model, numerical calculation results of the UMAT subroutine and experimental results.
2. The different rock inclinations have different effects on the ground on the left and right sides of the tunnel. When *α* is 0°, 45°, 90°, the ground horizontal displacement on the left and right sides is equivalent. The inclination of rock also has a greater impact on the ground settlement above the tunnel. As the rock inclination angle *α* gradually increases from 0° to 90°, the ground settlement first decreases and then slowly increases. When *α* is 0°, the ground settlement is the largest (12.4 mm). When *α* is 45°, the ground settlement is the smallest (11.1 mm).
3. The different rock inclinations also have different effects on the convergence of the tunnel excavation section. The deformation of the vault is the most obvious, followed by the deformation of the arch bottom. The convergence values of them continuously decrease with the increase of the rock inclination, and their maximum convergence values are 23.4 mm and 17.3 mm, respectively. The deformation of the arch waist and arch shoulder is relatively small, but the convergence values of the them has the opposite trends with the inclination of the rock layer. When *α* is 0°, the convergence value of the arch waist is the largest (3.5 mm). When *α* is 15°, the convergence value of the arch shoulder is the largest (2.0 mm).

## Data Availability

The data in this paper are obtained according to theoretical derivation, experimental testing and numerical simulation. The subject needs further research and is inconvenient to be disclosed. The data that support the findings of this study are available from the corresponding author upon reasonable request.
